# Common variation at 12q24.13 (*OAS3*) influences chronic lymphocytic leukemia risk

**DOI:** 10.1038/leu.2014.311

**Published:** 2014-12-05

**Authors:** G P Sava, H E Speedy, M C Di Bernardo, M J S Dyer, A Holroyd, N J Sunter, H Marr, L Mansouri, S Deaglio, L Karabon, I Frydecka, K Jamroziak, D Woszczyk, G Juliusson, K E Smedby, S Jayne, A Majid, Y Wang, C Dearden, A G Hall, T Mainou-Fowler, G H Jackson, G Summerfield, R J Harris, A R Pettitt, D J Allsup, J R Bailey, G Pratt, C Pepper, C Fegan, R Rosenquist, D Catovsky, J M Allan, R S Houlston

**Affiliations:** 1 Division of Genetics and Epidemiology, The Institute of Cancer Research, Sutton, UK; 2 The Ernest and Helen Scott Haematological Research Institute, Department of Biochemistry and Department of Cancer Studies and Molecular Medicine, University of Leicester, Leicester, UK; 3 Northern Institute for Cancer Research, Newcastle University, Newcastle upon Tyne, UK; 4 Department of Immunology, Genetics and Pathology, Science for Life Laboratory, Uppsala University, Uppsala, Sweden; 5 Department of Medical Sciences and Human Genetics Foundation, University of Turin, Turin, Italy; 6 Department of Experimental Therapy, Institute of Immunology and Experimental Therapy, Polish Academy of Sciences, Wroclaw, Poland; 7 Department and Clinic of Urology, Wroclaw Medical University, Wroclaw, Poland; 8 Department of Hematology, Institute of Hematology and Transfusion Medicine, Warsaw, Poland; 9 Department of Haematology, State Hospital, Opole, Poland; 10 Lund Strategic Research Center for Stem Cell Biology and Cell Therapy, Hematology and Transplantation, Lund University, Lund, Sweden; 11 Unit of Clinical Epidemiology, Department of Medicine, Karolinska Institutet, Stockholm, Sweden; 12 Medical Research Council Toxicology Unit, Leicester University, Leicester, UK; 13 Haemato-Oncology, Division of Pathology, The Institute of Cancer Research, Sutton, UK; 14 Haematological Sciences, Medical School, Newcastle University, Newcastle upon Tyne, UK; 15 Department of Haematology, Royal Victoria Infirmary, Newcastle upon Tyne, UK; 16 Department of Haematology, Queen Elizabeth Hospital, Gateshead, Newcastle upon Tyne, UK; 17 Department of Molecular and Clinical Cancer Medicine, University of Liverpool, Liverpool, UK; 18 Department of Haematology, Hull Royal Infirmary, Hull, UK; 19 Hull York Medical School and University of Hull, Hull, UK; 20 Department of Haematology, Birmingham Heartlands Hospital, Birmingham, UK; 21 Department of Haematology, School of Medicine, Cardiff University, Cardiff, UK; 22 Cardiff and Vale National Health Service Trust, Heath Park, Cardiff, UK

Chronic lymphocytic leukemia (CLL) is the most common form of lymphoid malignancy in Western countries^1^. Recent multi-stage genome-wide association studies (GWAS) have shown that part of the eight-fold increased risk of CLL seen in first-degree relatives of patients can be ascribed to the co-inheritance of multiple low-risk variants.^2, 3, 4, 5, 6^

Current projections for the number of independent regions harbouring common variants that are associated with CLL suggest that additional risk loci conferring modest effects should be identified by the expansion of discovery GWAS data sets.^2^

In this study, we have made use of a meta-analysis of GWAS data, followed by validation in multiple independent case–control series, to identify a novel susceptibility locus for CLL at 12q24.13.

The discovery phase comprised two previously described GWAS conducted in the United Kingdom^2, 5^ (see Supplementary Methods). UK-GWAS-1; 517 CLL cases (155 enriched for genetic susceptibility by virtue of family history) genotyped using Illumina HumanCNV370-Duo BeadChips^5^ and 2698 controls from the Wellcome Trust Case Control Consortium 2 (WTCCC2) 1958 Birth cohort, typed using Hap1.2M-Duo Custom array.^7^ UK-GWAS-2; 1271 CLL cases genotyped using the Illumina Omni Express BeadChip and 2501 UK Blood Service Donor controls typed using Hap1.2M-Duo Custom arrays.^2^ To harmonise GWAS data sets we recovered untyped genotypes by imputation using IMPUTEv2 with 1000genomes as a reference (phase 1 integrated variant set (b37) from March 2012) (Supplementary Methods). Genomic control lambda values for UK-GWAS1 and UK-GWAS2 were 1.04 and 1.05, respectively, thereby excluding significant differential genotyping or cryptic population substructure.^2^

Post quality control the two GWAS provided data on 1739 cases and 5199 controls. In a meta-analysis we identified 156 common SNPs (minor allele frequency>0.01), typed in either UK GWAS-1 or 2, that showed good evidence of an association (ie *P*<1.5 × 10^−4^) and did not map to any of the 30 loci that have previously been associated with CLL risk.^2^

Seven SNPs chosen on the basis of strength of association and/or biological plausibility of the annotated gene (that is, a role in B-cell or cancer biology) were genotyped in the UK replication series (Supplementary Table 1), which comprised 1195 CLL cases ascertained from an ongoing national study being conducted by the Institute of Cancer Research and 2568 controls ascertained through the National Study of Colorectal Cancer^8^ (Supplementary Methods).

Two SNPs, rs10735079 and rs17512800, provided further evidence for an association with CLL risk (ie *P*<0.05) and these two SNPs were taken forward for genotyping in a further replication series from Sweden, which comprised 347 CLL cases and 342 controls (Supplementary Table 1). This case control analysis provided additional evidence for an association between rs10735079 and CLL risk. Subsequently we genotyped rs10735079 in three further case–control series, Poland-1 (105 cases, 101 controls), Poland-2 (176 cases, 209 controls) and Italy (186 cases, 155 controls) (Supplementary Methods). In the combined analysis of all series the association between rs10735079 and CLL attained genome-wide significance (combined OR per allele=1.18, 95% CI:1.12–1.26, *P*=2.34 × 10^−8^) (Figure 1). The association was not restricted to *IGHV* mutation and showed no relationship with either sex or age (Supplementary Table 2).

rs10735079 maps to intron 2 of the 2′-5′-oligoadenylate synthetase 3 (*OAS3*) gene, one of three *OAS* genes clustering at 12q24.13 (Figure 2), and is in LD (*r*^2^=0.87) with the splice acceptor variant of *OAS1*, rs10774671, which mediates alternative splicing of *OAS1* transcription and affects enzymatic activity.^9^ Although attractive as the basis of the 12q24.13 association the association with CLL is stronger for rs10735079 than rs10774671 (*P*=1.16 × 10^−5^ and 1.74 × 10^−4^, respectively; Supplementary Table 3).

The significant dose relationship between rs10735079 genotype and *OAS3* expression in blood, with the risk allele being associated with reduced levels of mRNA (*P*=5.4 × 10^−29^; Supplementary Table 4), supports a role for rs10735079 genotype mediating its effect on CLL through differential *OAS3* expression rather than impacting on *OAS1*.

Although rs10735079 is not predicted to lie in an active promoter or strong enhancer element, the correlated SNP rs6489879 (*r*^2^=0.99) that maps to intron 1 of *OAS3* resides in a region predicted to be a strong enhancer in lymphoblastoid GM12878 cells and to be involved in binding of a number of transcription factors including IRF4 (interferon regulatory factor-4), a lymphocyte-specific transcription factor (Figure 2; Supplementary Table 3).

OAS is induced by interferon in response to viral infection activating 2-5A-dependent RNase L degradation of viral RNA^10^ and variation in OAS genes has been reported to be a determinant of viral susceptibility.^9, 11, 12, 13^ Given the possible role of viral response in the pathogenesis of CLL, although speculative, it is therefore possible that genetic variation in *OAS3* influences risk of developing CLL through differing response to antigenic challenge. Moreover, *OAS3* is a B-cell receptor (BCR) signature gene.^14^ Intriguingly as variation in the BCR genes *IRF4* (ref. 5), *BCL2* (ref. 3) and *HLA-DQA1* (ref. 15) has previously been implicated by GWAS as determinants of CLL risk this suggests a common aetiological pathway through differential BCR-activation.

Although further functional studies are required to fully elucidate the biological basis of the 12q24.13 association, our finding brings the total number of risk loci identified for CLL thus far to 31 and provides additional support for the role of inherited genetic factors in the aetiology of CLL.

URLS

Blood eQTL browser: http://genenetwork.nl/bloodeqtlbrowser/

PLINK: http://pngu.mgh.harvard.edu/~purcell/plink/

Illumina: http://www.illumina.com/

Kaspar: http://www.lgcgenomics.com/genotyping/kasp-genotyping-chemistry/

SNAP: http://www.broadinstitute.org/

Haploreg: http://www.broadinstitute.org/mammals/haploreg/haploreg.php

visPIG-Visual Plotting Interface for Genetics: http://vispig.icr.ac.uk/

## Figures and Tables

**Figure 1:**
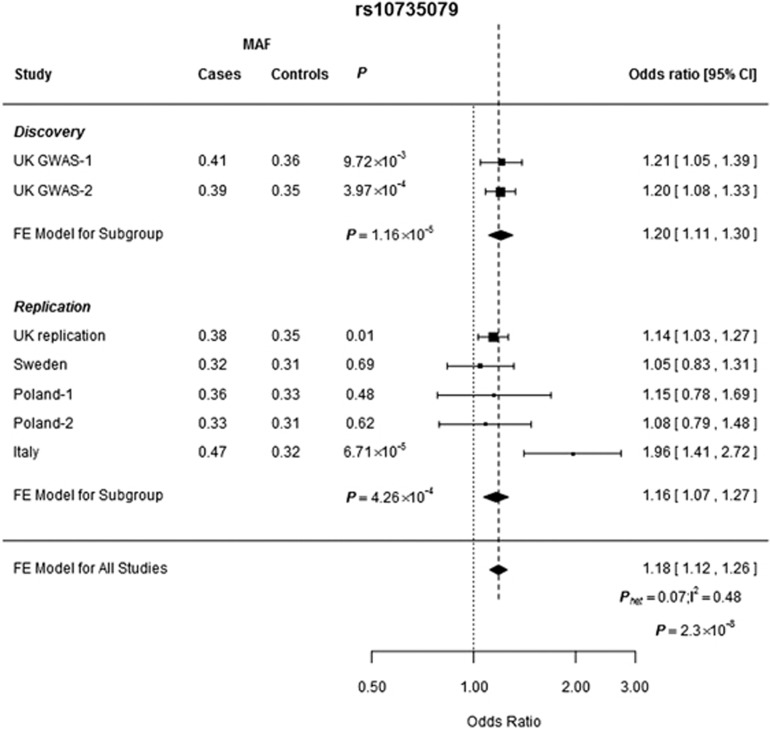
Forest plot of the ORs for the association between CLL and rs10735079. Studies were weighted according to the inverse of the variance of the log of the OR calculated by unconditional logistic regression. Horizontal lines: 95% CI. Box: OR point estimate; box area is proportional to the weight of the study. Diamond (and broken line): overall summary estimate, with CI given by its width. Unbroken vertical line: null value (OR=1.0). FE, fixed effects; MAF, minor allele frequency.

**Figure 2:**
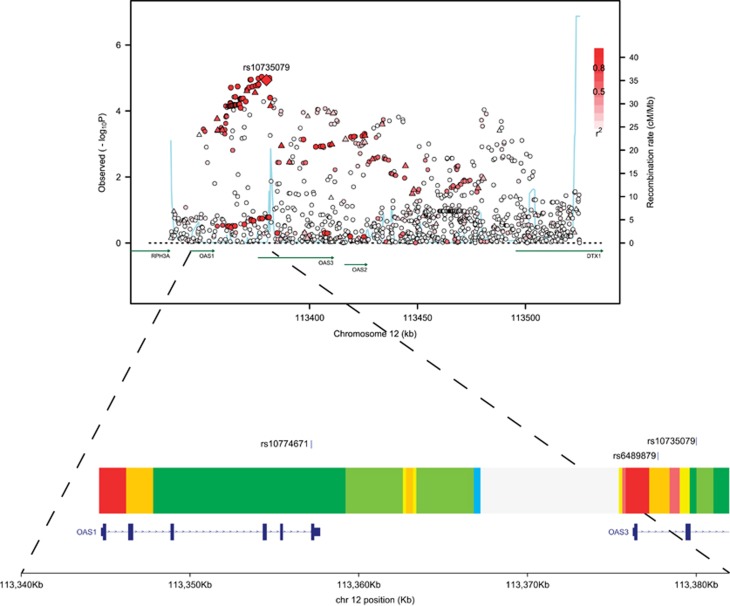
Regional plot of association results, recombination rates and chromatin state segmentation track for 12q24.13 susceptibility locus. Association results of both genotyped (triangles) and imputed (circles) SNPs in the GWAS samples and recombination rates. −log_10_ *P-*values (*y* axis) of the SNPs are shown according to their chromosomal positions (*x* axis). rs10735079 is shown as a large diamond and is labelled by its rsID. Colour intensity of each symbol reflects the extent of LD with the top genotyped SNP; white (*r*^2^=0) through to dark red (*r*^2^=1.0) Genetic recombination rates, estimated using HapMap Utah residents of Western and Northern European ancestry (CEU) samples, are shown with a light blue line. Physical positions are based on NCBI Build 37 of the human genome. Also shown are the relative positions of genes and transcripts mapping to the region of association. Genes have been redrawn to show the relative positions; therefore, maps are not to physical scale. The lower panel shows the chromatin state segmentation track (ChromHMM) for LCL data derived from the ENCODE project and the positions of SNPs of interest (produced using visPIG-Visual Plotting Interface for Genetics).
