# Supplementary material for: Common variation at 12q24.13 (OAS3) influences chronic lymphocytic leukemia risk
Source: Leukemia. 2014 Dec 5;29(3):748–51. doi: 10.1038/leu.2014.311 (PMC4360210; doi:10.1038/leu.2014.311)
Supplement: Supplementary Table Legends [file leu2014311x5.doc]

**SUPPLEMENTARY FILE LEGENDS**

**Supplementary Table 1: Relationship between rs10735079 genotype and age at diagnosis, sex and *IGHV* status in the UK-GWAS data.**

**Supplementary Table 2: Summary of genetic annotation by Haploreg with GWAS *P* values for proxies of rs10735079.** Data are shown for proxy SNPs of rs10735079 (r2>0.8 in 1000Genomes EUR phase 1 data) that have dbSNP annotations of missense, synonymous or splice acceptor, or that demonstrate evidence of histone marks, DNAse hypersensitivity sites or transcription factor occupancy in HaploRegv2 analysis of ENCODE data generated on the GM12878 cell line. P values from the UK-GWAS meta-analysis are presented along with typed/imputed status.

**Supplementary Table 3: Association between rs10735079 and rs10774671 and transcript levels of cis-genes in the Blood eQTL browser (FDR 0.5).** FDR= false discovery rate.
