# Supplementary material for: Common variation at 12q24.13 (OAS3) influences chronic lymphocytic leukemia risk
Source: Leukemia. 2014 Dec 5;29(3):748–51. doi: 10.1038/leu.2014.311 (PMC4360210; doi:10.1038/leu.2014.311)
Supplement: Supplementary Methods [file leu2014311x6.doc]

**SUPPLEMEMTARY METHODS**

**Participants**

Data collection from participants was approved by the respective institutional review boards, and all participants provided written informed consent in accordance with the Declaration of Helsinki. For all cases, the diagnosis of CLL had been confirmed in accordance with World Health Organization guidelines[^1^](#_ENREF_1).

**Discovery datasets**

The discovery phase comprised two previously described GWAS conducted in the UK. UK-GWAS-1; 517 CLL cases (155 enriched for genetic susceptibility by virtue of family history) genotyped using Illumina HumanCNV370-Duo BeadChips[^2^](#_ENREF_2) and 2,698 controls from the Wellcome Trust Case Control Consortium 2 (WTCCC2) 1958 Birth cohort, typed using Hap1.2M-Duo Custom array[^3^](#_ENREF_3). UK-GWAS-2; 1,271 CLL cases genotyped using the Illumina Omni Express BeadChip and 2 501 UK Blood Service Donor controls typed using Hap1.2M-Duo Custom arrays[^4^](#_ENREF_4). *λ*_GC_ values for UK-GWAS1 and UK-GWAS2 were 1.04 and 1.05, respectively, thereby excluding significant differential genotyping or cryptic population substructure[^4^](#_ENREF_4). Any ethnic outliers or individuals identified as related were excluded[^4^](#_ENREF_4). To harmonise GWAS datasets we made use of 1000genomes imputation (phase 1 integrated variant set (b37) from March 2012) using IMPUTEv2. Imputed data were analysed using SNPTEST v2 to account for uncertainties in SNP prediction. Association meta-analyses included only markers with info scores >0.4, Hardy-Weinberg equilibrium *P* > 10^−5^ and missingness rate <0.05[^4^](#_ENREF_4).

**Replication series**

For validation we made use of five additional case-control series. UK-replication; 1,195 CLL cases (707 male, mean age at diagnosis 62 years, SD ± 11) ascertained from an ongoing national study being conducted by the Institute of Cancer Research and 2,568 controls (1 053 male, mean age 64 years, SD ± 10) ascertained through the National Study of Colorectal Cancer[^5^](#_ENREF_5). Sweden; 347 CLL cases (241 male, mean age 63 years, SD ± 9) and 342 healthy controls (251 male, mean age 61 years, SD ± 7). Poland-1; 105 CLL cases (68 male, mean age 60 years SD ± 11) and 101 healthy controls (41 male, mean age 60 years SD ± 12). Poland-2; 176 CLL patients (100 male, mean age 64 years SD ± 11) and 209 healthy controls (137 male, mean age 31 years SD ± 9). Italy; 186 CLL patients (135 male, mean age 64 years SD ± 10) and 155 controls (119 male, mean age 44 years SD ± 16).Genotyping was conducted using competitive allele-specific PCR KASPar chemistry (LGC Genomics Ltd.). To confirm genotyping accuracy, duplicate samples were genotyped. To ensure the quality of genotyping in all assays, at least two negative controls and 1–2% duplicates (showing concordance >99.99%) were genotyped.

**Mutation status and expression quantitative trait loci (eQTL) analysis**

*IGHV* gene mutation status was determined for 666 UK, 330 Italian and 356 Swedish cases, as per BIOMED-2 protocols[^6^](#_ENREF_6). In accordance with published criteria[^7^](#_ENREF_7), we classified sequences with germline homology of ≥98% as unmutated and those with homology <98% as mutated. To examine the relationship between SNP genotype and gene expression, we made use of the data from the Blood eQTL browser resource[^8^](#_ENREF_8).

**Statistical and bioinformatic analysis**

Analyses were performed using R (version 3.0.2) and PLINK (version 1.07) software. The association between each SNP and CLL was assessed by the Cochran-Armitage trend test. Odds ratios (ORs) and 95% confidence intervals were calculated by unconditional logistic regression. Meta-analysis was conducted under a fixed-effects model. Associations by sex, age, and clinico-pathologic phenotypes were examined by logistic regression in case-only analyses. Linkage disequilibrium (LD) metrics were based on 1000 genomes pilot CEU data[^9^](#_ENREF_9). Haploreg[^10^](#_ENREF_10) was used to examine whether SNPs or their proxies (r^2^>0.8) annotate putative regulatory elements in lymphoblastoid GM12878 cells.

**REFERENCES**

1. Swerdlow SH, Campo E, Harris NL, Jaffe ES, Pileri SA, Stein H*, et al.* *WHO Classification of Tumours, Volume 2*, vol. 2. IARC Press, 2008.

2. Di Bernardo MC, Crowther-Swanepoel D, Broderick P, Webb E, Sellick G, Wild R*, et al.* A genome-wide association study identifies six susceptibility loci for chronic lymphocytic leukemia. *Nature genetics* 2008 Oct; **40**(10)**:** 1204-1210.

3. Power C, Elliott J. Cohort profile: 1958 British birth cohort (National Child Development Study). *International journal of epidemiology* 2006 Feb; **35**(1)**:** 34-41.

4. Speedy HE, Di Bernardo MC, Sava GP, Dyer MJ, Holroyd A, Wang Y*, et al.* A genome-wide association study identifies multiple susceptibility loci for chronic lymphocytic leukemia. *Nature genetics* 2014 Jan; **46**(1)**:** 56-60.

5. Penegar S, Wood W, Lubbe S, Chandler I, Broderick P, Papaemmanuil E*, et al.* National study of colorectal cancer genetics. *Br J Cancer* 2007; **97**(9).

6. van Krieken JH, Langerak AW, Macintyre EA, Kneba M, Hodges E, Sanz RG*, et al.* Improved reliability of lymphoma diagnostics via PCR-based clonality testing: report of the BIOMED-2 Concerted Action BHM4-CT98-3936. *Leukemia : official journal of the Leukemia Society of America, Leukemia Research Fund, UK* 2007 Feb; **21**(2)**:** 201-206.

7. van Krieken JHJM, Langerak AW, Macintyre EA, Kneba M, Hodges E, Sanz RG*, et al.* Improved reliability of lymphoma diagnostics via PCR-based clonality testing: [mdash] Report of the BIOMED-2 Concerted Action BHM4-CT98-3936. *Leukemia : official journal of the Leukemia Society of America, Leukemia Research Fund, UK* 2006; **21**(2).

8. Westra HJ, Peters MJ, Esko T, Yaghootkar H, Schurmann C, Kettunen J*, et al.* Systematic identification of trans eQTLs as putative drivers of known disease associations. *Nature genetics* 2013 Oct; **45**(10)**:** 1238-1243.

9. Johnson AD, Handsaker RE, Pulit SL, Nizzari MM, O'Donnell CJ, de Bakker PIW. SNAP: a web-based tool for identification and annotation of proxy SNPs using HapMap. *Bioinformatics* 2008 December 15, 2008; **24**(24)**:** 2938-2939.

10. Ward LD, Kellis M. HaploReg: a resource for exploring chromatin states, conservation, and regulatory motif alterations within sets of genetically linked variants. *Nucleic Acids Res* 2012 Jan; **40**(Database issue)**:** D930-934.
